# Critical appraisal of tubular putative eumetazoans from the Ediacaran Weng'an Doushantuo biota

**DOI:** 10.1098/rspb.2015.1169

**Published:** 2015-08-07

**Authors:** John A. Cunningham, Kelly Vargas, Liu Pengju, Veneta Belivanova, Federica Marone, Carlos Martínez-Pérez, Manuel Guizar-Sicairos, Mirko Holler, Stefan Bengtson, Philip C. J. Donoghue

**Affiliations:** 1 School of Earth Sciences, University of Bristol, Life Sciences Building, Tyndall Avenue, Bristol BS8 1TQ, UK; 2 Department of Palaeobiology and Nordic Center for Earth Evolution, Swedish Museum of Natural History, Stockholm 10405, Sweden; 3 Institute of Geology, Chinese Academy of Geological Sciences, 26 Baiwanzhuang Road, Beijing 100037, People's Republic of China; 4 Swiss Light Source, Paul Scherrer Institute, Villigen 5232, Switzerland; 5 Department of Geology, University of Valencia, C/Dr. Moliner 50, Burjassot, Valencia 46100, Spain

**Keywords:** Doushantuo, Ediacaran, tubular fossils, exceptional fossilization

## Abstract

Molecular clock analyses estimate that crown-group animals began diversifying hundreds of millions of years before the start of the Cambrian period. However, the fossil record has not yielded unequivocal evidence for animals during this interval. Some of the most promising candidates for Precambrian animals occur in the Weng'an biota of South China, including a suite of tubular fossils assigned to *Sinocyclocyclicus*, *Ramitubus*, *Crassitubus* and *Quadratitubus*, that have been interpreted as soft-bodied eumetazoans comparable to tabulate corals. Here, we present new insights into the anatomy, original composition and phylogenetic affinities of these taxa based on data from synchrotron radiation X-ray tomographic microscopy, ptychographic nanotomography, scanning electron microscopy and electron probe microanalysis. The patterns of deformation observed suggest that the cross walls of *Sinocyclocyclicus* and *Quadratitubus* were more rigid than those of *Ramitubus* and *Crassitubus*. *Ramitubus* and *Crassitubus* specimens preserve enigmatic cellular clusters at terminal positions in the tubes. Specimens of *Sinocyclocyclicus* and *Ramitubus* have biological features that might be cellular tissue or subcellular structures filling the spaces between the cross walls. These observations are incompatible with a cnidarian interpretation, in which the spaces between cross walls are abandoned parts of the former living positions of the polyp. The affinity of the Weng'an tubular fossils may lie within the algae.

## 1. Introduction

Molecular clock analyses estimate that metazoans diversified in the Ediacaran or Cryogenian tens or hundreds of millions of years before the beginning of the Cambrian [1,2]. While the fossil record is undoubtedly incomplete, no fossil data unequivocally support these predictions. Fossils from the classical Ediacaran biota are notoriously difficult to interpret. Based on the available evidence, some ediacarans might best be interpreted as total-group animals [3–5], but these interpretations are not sufficiently robust to substantiate the presence of crown-group animals at this time. The Weng'an biota [6] from the Doushantuo Formation of South China has yielded a suite of Early Ediacaran microfossils described as embryonic [7] and adult [8] animals, including putative stem-group metazoans [9], sponges [10,11], cnidarians [12] and bilaterians [8,13,14]. These are found alongside fossils assigned to acritarchs [15,16] and algae [17]. However, all of the claims for animal affinity have been contested [18–23], and none can currently be considered to represent undisputed animal fossils [24]. A suite of tubular fossils [25–27] from the Doushantuo, assigned to the genera *Sinocyclocyclicus*, *Ramitubus*, *Crassitubus* and *Quadratitubus*, are rare examples of widely accepted Precambrian metazoans [28–30]. These fossil taxa have been interpreted as a closely interrelated group of soft-bodied animals comparable to tabulate corals, and they have been marshalled as evidence for cnidarian-like eumetazoans at this time [19,25]. However, alternative interpretations have been suggested for these tubular taxa, including algae [31,32] and cyanobacteria [27]. Testing among these competing interpretations requires the resolution of key uncertainties concerning the anatomy of the tubular taxa, including the nature of the cross walls, critical to the cnidarian interpretation, and whether their internal compartments represent the boundaries between cells or between some other kind of structure. To this end, we use synchrotron radiation X-ray tomographic microscopy (SRXTM), ptychographic X-ray computed tomography (PXCT), imaging of back-scattered electrons (BSE) and electron probe microanalysis (EPMA) to elucidate the anatomy and original composition of these tubular taxa. We assess the implications of these new data for understanding their phylogenetic affinity.

## 2. Material and methods

Specimens were extracted from rock samples from the Upper Phosphorites of the Datang Quarry, Weng'an, Guizhou Province, China (see [16] for a description of the stratigraphic context). The carbonate constituents of the samples were dissolved in *ca* 10% acetic acid and the apatite-preserved fossils were recovered from the resulting residues by manual sorting under a binocular microscope. Figured specimens are housed at the Swedish Museum of Natural History, Stockholm (X 5322–X 5330). All tomographic measurements were carried out at the Swiss Light Source, Paul Scherrer Institute, Switzerland. The SRXTM experiments [33,34] were conducted at the X02DA (TOMCAT) beamline and the PXCT experiments [35] at the X12SA (cSAXS) beamline [35]. All specimens were examined using SRXTM. Images were recorded at 1501 stepwise increments through a rotation of 180° at a beam energy of around 17.5 keV. Specimens were scanned with a 20× objective, resulting in voxel dimensions of 0.325–0.37 µm. The projection images were processed and rearranged into dark- and flatfield-corrected sinograms, after which they were reconstructed using a gridding procedure and a highly optimized routine based on the Fourier transform method [36]. One specimen (X 5324) was also examined using PXCT, which enables nanoscale-resolution tomography. The PXCT measurement was carried out at 8.7 keV photon energy using an interferometrically controlled three-dimensional scanning stage [37], the sample was scanned across a coherent X-ray beam with a field of view of 150 × 15 µm at 360 angular orientations that spanned a range from 0° to 180°. At each scanning point, diffraction patterns were measured with an Eiger detector [38] with a 0.1 s exposure time. Ptychographic reconstructions were carried out using the difference map algorithm with a maximum-likelihood refinement [39,40], and tomographic synthesis was carried out with projection alignment and post-processing as described in [41,42]. The PXCT data have voxel dimensions of 0.044 µm. All tomographic data were analysed using Avizo software. Two specimens (X 5326 and X 5330) were examined using BSE and EPMA using the methods described in [23]. We selected examples from each of the four tubular genera described in detail by Liu *et al.* [25], which are primarily distinguished by differences in overall morphology, in the nature of their cross walls, and in the presence or absence of a sheath.

## 3. Results

### (a) Sinocyclocyclicus

**Figure 1.**
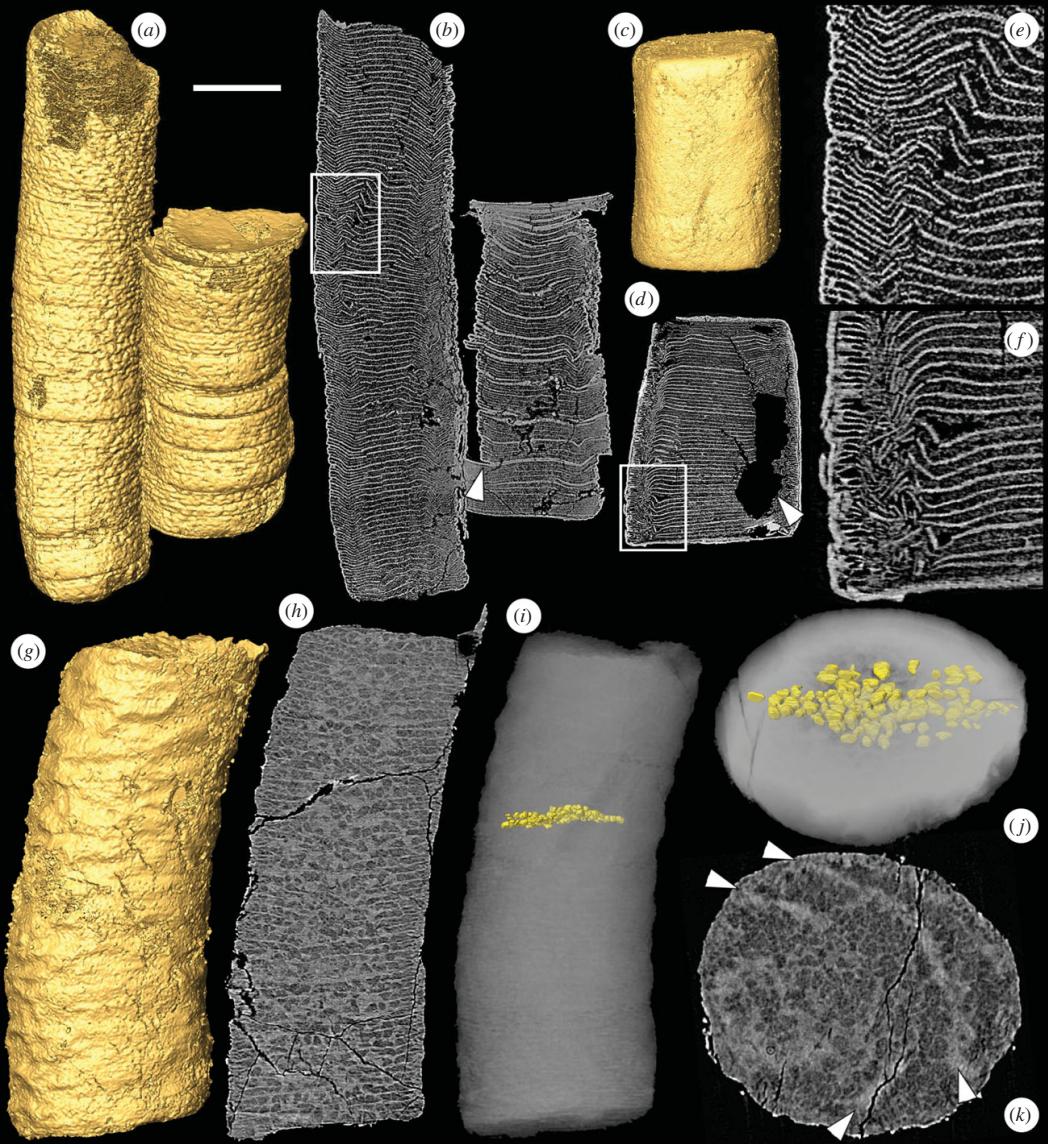
SRXTM images of the tubular fossils *Sinocyclocyclicus* and *Quadratitubus*. (*a*) SRXTM surface model of *Sinocyclocyclicus*, X 5322; (*b*) longitudinal SRXTM slice through the specimen in (*a*) showing brittle deformation of the cross walls, the arrowhead indicates displacement along the plane of the cross wall, the region in the box is enlarged in (*e*); (*c*) surface model of *Quadratitubus*, X 5323; (*d*) longitudinal SRXTM section through the specimen in (*c*) showing brittle deformation, the arrowhead indicates a void created by pyrite dissolution, the region in the box is enlarged in (*f*); (*e*) higher magnification image of boxed region in (*b*); (*f*) higher magnification image of boxed region in (*d*); (*g*) SRXTM surface model of a specimen of *Sinocyclocyclicus* with ovoid to sub-cuboid structures preserved in the spaces between the cross walls, X 5324; (*h*) longitudinal SRXTM slice of the specimen in (*g*); (*i*) reconstruction based on PXCT data showing the arrangement of structures between two successive cross walls; (*j*) reconstruction in (*i*) shown in oblique view; (*k*) transverse SRXTM slice of the specimen in (*g*), the arrowheads indicate the positions of two successive cross walls. Scale bars: (*a*,*b*) 120 µm; (*c*,*d*) 143 µm; (*e*) 38 µm; (*f*) 40 µm; (*g*–*i*) 58 µm; (*j*,*k*) 32 µm. (Online version in colour.)

*Sinocyclocyclicus* consists of a straight, non-branching tube with a circular cross section. It has complete and incomplete cross walls that alternate with one another [25]. The cross walls are preserved in a high X-ray attenuation phase (figure 1), with similar chemical properties to those found in high-attenuation phases in other Doushantuo specimens by [23] (electronic supplementary material, figure S1*a*). Some of the *Sinocyclocyclicus* specimens that we have examined have numerous examples of brittle fracture of the cross walls (figure 1*a*,*b*,*e*; electronic supplementary material, figure S2*a*) as well as displacement along the plane of these cross walls (figure 1*b*, arrowhead). Examples of organic degradation (as inferred from cross walls that are truncated by irregular regions of void-filling cement) or of ductile deformation without fracture are rarely observed in this taxon.

Our SRXTM and PXCT data reveal previously unknown features in the anatomy of *Sinocyclocyclicus* that occupy spaces between the cross walls (figure 1*g*–*k*). These regular structures are ovoid to sub-cuboid in shape and measure around 5–10 µm in diameter. The interiors of the structures are mineralized in a low X-ray attenuation phase that is typical of soft-tissue preservation [23,43], surrounded by a high-attenuation phase. In the central part of one specimen, a single row of these structures fills the space between successive complete cross walls (figure 1*h*–*k*). Manual computed tomographic characterization of the three-dimensional morphology of the component structures between adjacent cross walls (figure 1*i*–*j*) based on the high-resolution PXCT data shows their regular size and shape. Towards the edges, smaller structures *ca* 2 µm in diameter fill the spaces between the incomplete cross walls (figure 1*h*).

### (b) Quadratitubus

*Quadratitubus* is similar to *Sinocyclocyclicus* in having a straight non-branching form and alternating complete and incomplete cross walls. It differs, however, in having a quadrangular (rather than approximately circular) cross section [25]. *Quadratitubus* shows a similar pattern of deformation to *Sinocyclocyclicus*. Brittle deformation is common (figure 1*c*,*d*,*f*), but ductile deformation without fracture is rarely observed. Likewise, evidence for organic degradation of the cross walls is rare (the void in the specimen in figure 1*c*,*d*,*f* is the result of a pyrite trail). Our three-dimensional data support the anatomical description presented by Liu *et al.* [25] and do not provide additional information.

### (c) Ramitubus

**Figure 2.**
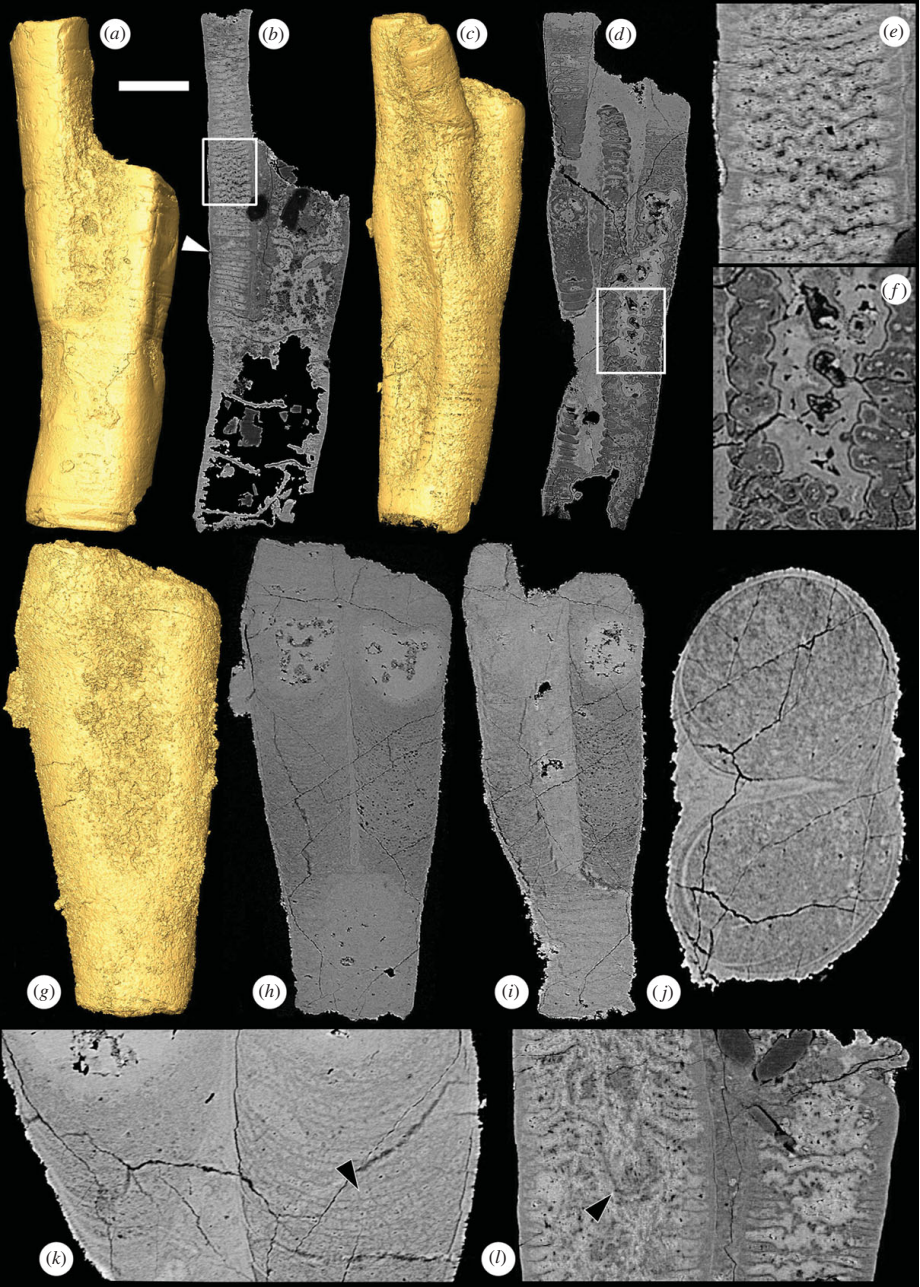
SRXTM images of the tubular fossil *Ramitubus*. (*a*) SRXTM surface model of *Ramitubus*, X 5325, showing ductile deformation of the tube; (*b*) longitudinal SRXTM section through the specimen in (*a*) showing ductile deformation of the crosswalls, the arrowhead indicates a region where there are several successive compartments of lower than average height, the region in the box is enlarged in (*e*); (*c*) SRXTM surface model of *Ramitubus*, X 5326; (*d*) longitudinal SRXTM section through the specimen in (*c*) showing organic degradation of the cross walls, the region in the box is enlarged in (*f*); (*e*) higher magnification image of boxed region in (*b*); (*f*) higher magnification image of boxed region in (*d*) showing cross walls truncated by an irregular region of void-filling cement; (*g*) SRXTM surface model of *Ramitubus*, X 5327; (*h*) longitudinal SRXTM section through the specimen in (*g*), note the high-attenuation (bright), nearly spherical structures towards the end of each branch; (*i*) longitudinal section through the specimen in (*g*) at a different level, note the change in the pattern of the cross walls after branching; (*j*) transverse SRXTM section through the specimen in (*g*); (*k*) higher magnification SRXTM section of the specimen in (*g*) showing finer layers between the cross walls, the arrowhead shows a region where these layers are made up of sub-angular structures; (*l*) longitudinal SRXTM section through the *Ramitubus* specimen in (*a*), the arrowhead indicates down-folding of a cross wall that results in a prominent pocket-like space towards the centre of one of the specimen. Scale bars: (*a*,*b*) 183 µm; (*c*,*d*) 126 µm; (*e*) 48 µm; (*f*) 40 µm; (*g*–*i*) 89 µm; (*j*) 36 µm; (*k*) 36 µm; (*l*) 64 µm. (Online version in colour.)

*Ramitubus* (figure 2) has a circular cross section and it is the only one of the four tubular genera to display branching. It is also distinct in that it has complete cross walls only, which is in contrast to the alternating complete and incomplete walls seen in the other taxa [25]. The spacing between the cross walls is typically 10–15 µm, but some regions exhibit a series of compartments of lower than average height (*ca* 7 µm) in a row (figure 2*b*, arrowhead). *Ramitubus* shows abundant evidence of both ductile deformation of specimens (figure 2*a*) and cross walls (figure 2*b*,*e*), and for organic degradation (figure 2*c*,*d*,*f*; electronic supplementary material, figures S1*b* and S2*b*). We found no evidence of brittle deformation of cross walls, though an incomplete transverse separation associated with ductile deformation in one specimen indicates tearing of the outer wall (electronic supplementary material, figure S3). In addition to the deformation observed, there are some prominent pocket-like structures around 50 µm deep created by down-folding of the central regions of some of the cross walls (figure 2*l*, arrowhead). Specimens can be deformed such that the cross section is up to twice as long as broad (electronic supplementary material, figure S3). This suggests greater deformation than in *Sinocyclocyclicus* and *Quadratitubus*, which generally have cross sections that are close to the circular or quadrangular sections that they are assumed to have had in life (figure 1*c*,*k*).

After branching, the internal structure can change (figure 2*g–k*). In one specimen, the cross walls become concave upward in the region after the branching point (figure 2*h*,*i*,*k*), rather than straight and perpendicular to the tube length. Here, the cross walls are mainly preserved in a low-attenuation phase, especially towards the centre of the specimen and towards the ends of the branches (figure 2*h*,*i*,*k*); this contrasts with other regions of the specimen where they are preserved in a high-attenuation phase (e.g. figure 2*k*, left hand margin). Between adjacent cross walls in the branches there are three to four finer walls, which are preserved in a low-attenuation phase. In some regions, the spaces between the finer walls are filled by a single layer of sub-angular structures (figure 2*k*, arrowhead). Towards the ends of both branches of the same specimen, there are nearly spherical regions that are filled with high-attenuation void-filling mineral growth (figure 2*h*). These are approximately 100 µm in diameter in each of the branches and lie around 50–100 µm from the end of the tube in each case. The structures clearly represent the position of former voids that have been filled by diagenetic mineralization. The similar size, shape and position of the structures in the two tubes suggest that these voids might preserve the topology of structures of a biological origin. This is also supported by the similarities in size, shape and position to structures in *Crassitubus* (discussed below). The similarities between the structures in the two tubes suggest that they were formed in a coordinated way.

### (d) Crassitubus

**Figure 3.**
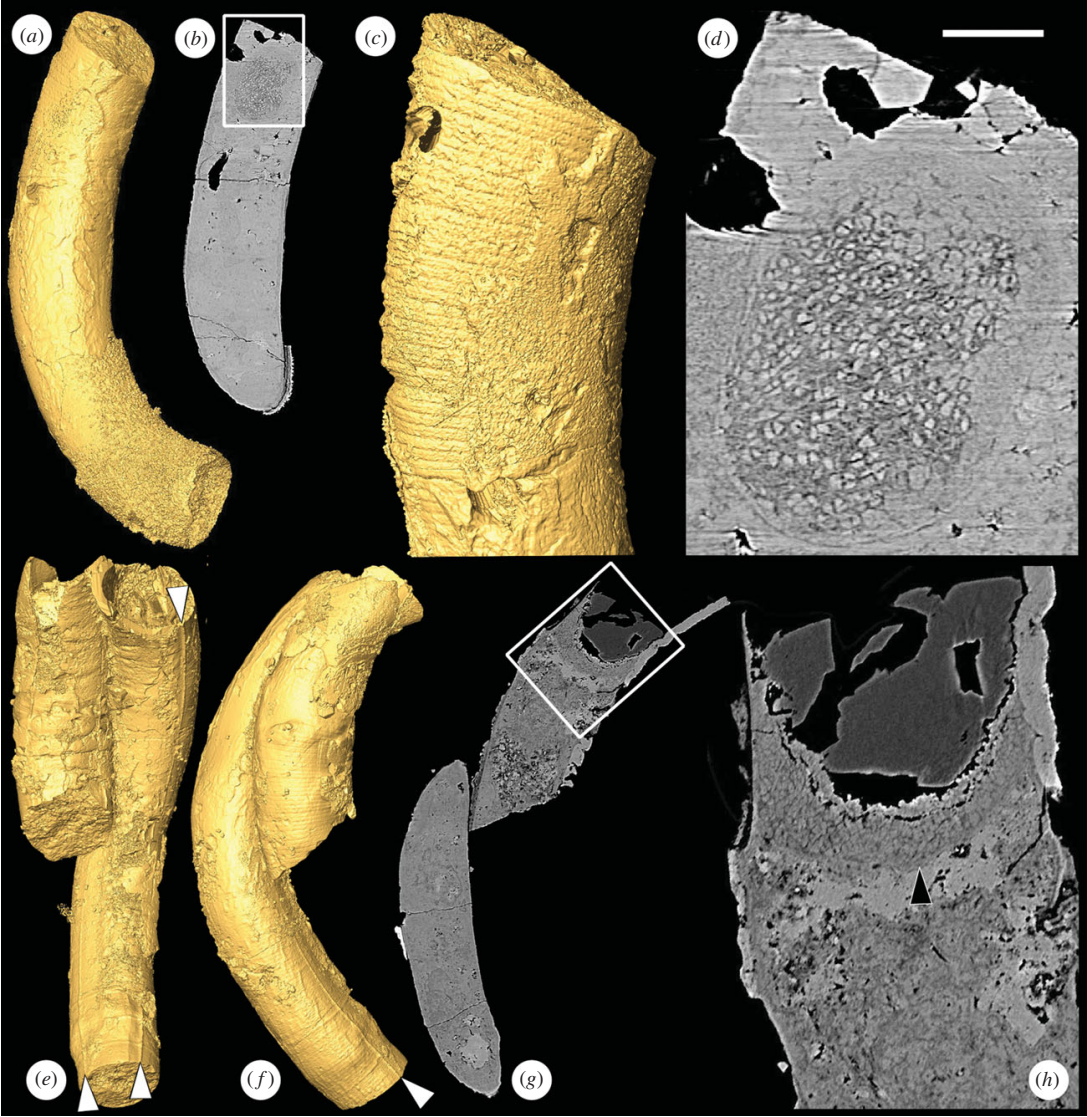
SRXTM images of the tubular fossil *Crassitubus*. (*a*) SRXTM surface model of *Crassitubus* specimen X 5328; (*b*) longitudinal SRXTM slice through the specimen in (*a*), the region in the box is enlarged in (*d*); (c) SRXTM surface model of the specimen in (*a*) showing the exterior of the specimen in the region of the structure shown in (*d*), note that the former positions of cross walls are visible; (*d*) higher magnification image of boxed region in (*g*) showing a nearly spherical structure composed of sub-angular objects close to the end of the tube; (*e*) SRXTM surface model of *Crassitubus* specimen X 5329; (*f*) SRXTM surface model of the specimen in (*e*) in a different orientation; (*g*) SRXTM slice through the specimen in (*e*), the boxed region is enlarged in (*h*); (*h*) higher magnification image of the boxed region in (*g*), arrowhead indicates a similar structure to that shown in (*d*). Scale bars: (*a*,*b*) 137 µm; (*c*) 62 µm; (*d*) 26 µm; (*e–g*) 200 µm; (*h*) 50 µm. (Online version in colour.)

*Crassitubus* is characterized by its curved, non-branching form as well as by an enveloping sheath that bears a longitudinal ridge (figure 3). Our data show that some specimens have at least two longitudinal ridges, rather than the single ridge seen in previously described material (figure 3*e*). Like *Ramitubus*, *Crassitubus* frequently exhibits evidence of organic decay and ductile deformation, and can be twisted and knotted [25]. There is no evidence of brittle fracture. In one of the specimens, there is an ovoid structure at one end of the tube that measures approximately 100 µm in the maximum dimension (figure 3*a*–*d*). It is composed of facetted structures that are up to 5 µm in diameter and that in some instances are organized into diads or tetrads, suggesting that they were formed by division. For these reasons, we interpret the structures as cellular compartments. It is possible that this is a coincidental, and not a biological, association between the cellular clusters and the *Crassitubus* organism, particularly as no internal cross walls are visible in this specimen, suggesting that it might be poorly preserved. The original positions of cross walls are visible on the outside of the specimen, both in the region of the cluster (figure 3*c*) and elsewhere. These walls must have been incomplete if they were to have allowed space for the cell cluster (such a pattern of cross walls is seen in fig. 2d of [19]). However, the presence of a similar structure in the same position of a second *Crassitubus* specimen (figure 3*e–h*, arrowhead in figure 3*h*) gives some weight to the argument that the structures are part of the organism's biology.

## 4. Evidence for original composition

We observed numerous examples of brittle deformation in *Sinocyclocyclicus* and *Quadratitubus*. As Liu *et al.* [25] have observed, this does not necessarily indicate that the organism was mineralized in life. The fractures could have occurred after diagenetic mineralization of a soft tissue: brittle fracture is seen occasionally in the embryo-like fossils from Doushantuo, which are accepted to have been non-mineralized. Moreover, Liu *et al.* [25] have argued that the presence of twisted or knotted specimens and of organic degradation suggests that the tubular fossils were probably not mineralized in life. However, we observed only rare examples of either of these phenomena in *Sinocyclocyclicus* or *Quadratitubus*, but they were common in *Ramitubus* and *Crassitubus*, and all of the figured examples in Liu *et al.* [25] are from the latter two taxa (e.g. their pl. 2, fig. 3; pl. 6, figs 18–19, 21; pl. 7, fig. 16; pl. 8, figs 8–9 for deformed specimens and pl. 8, figs 6–7 for organic degradation). This contrast between the tubular genera indicates that the *Sinocyclocyclicus* and *Quadratitubus* specimens were prone to brittle fracture at some point when the other taxa were not. An interpretation that these taxa had different taphonomic or diagenetic histories is unlikely given that the fossils were recovered from the same unit of the Doushantuo Formation [25]. A second possibility is that *Sinocyclocyclicus* and *Quadratitubus* were mineralized earlier in diagenesis. This might be explained if these taxa had walls that were thicker or more rigid or if they were composed of a substance more readily predisposed to mineralization. A third possibility is that they were mineralized during life. The fact that rare specimens of *Sinocyclocyclicus* and *Quadratitubus* have ductile deformation of the cross walls indicates that they may have been non-mineralized in life. However, it is also possible that they were lightly biomineralized like taxa such as *Cloudina* and *Sinotubulites*, which also show evidence for both brittle and ductile deformation but are generally considered to have been mineralized to some extent in life [44]. In any case, it seems that the cross walls of *Sinocyclocyclicus* and *Quadratitubus* were more rigid than those of the other taxa.

## 5. Phylogenetic affinities

It seems likely that the four tubular genera are closely related to one another, although it is difficult to devise a definitive test of this hypothesis. *Sinocyclocyclicus* and *Quadratitubus* are particularly alike, having similar construction and mechanical properties. *Ramitubus* and *Sinocyclocyclicus* both have similar fine structures between the cross walls, indicating a similar mode of construction and perhaps a close phylogenetic relationship. *Crassitubus* has a distinctive sheath with longitudinal ridges on the exterior, but the similarities between its alternating complete and incomplete cross walls and those of *Sinocyclocyclicus* and *Quadratitubus* also suggest a close relationship.

The competing hypotheses of affinity for the tubular fossils make different predictions about what lies in the spaces between the cross walls. The first possibility to consider is that the structures between the cross walls are not an original aspect of the anatomy of the tubular organisms but, rather, taphonomic or diagenetic structures that mould the spaces between cross walls. There are a number of different processes that might create such moulds, but none provides a good interpretation of the structures observed. Clumps of degraded organic material occur in some Doushantuo specimens, but they do not have the regular and evenly layered arrangement of the structures in question. Similarly, void-filling apatite mineralization is common in the deposit [23] but cannot explain this regular pattern of structures between the cross walls. Hydrothermal veining can produce a pattern that is superficially similar to that observed in the fossils, but this explanation is hard to reconcile with the regularity of the structures or the finely crystalline nature of the interior of the structures, which is similar to that known to preserve cells in this deposit [23]. Fracture is common in *Sinocyclocyclicus* but it is not manifest in this kind of pattern. In addition, it would be expected that such veins would commonly cut the cross walls of the organism. While there are a couple of examples where these structures appear to continue over the cross walls (figure 1*h*), their rarity suggests that this might be better explained by chance alignment of biological structures. An interpretation of the low-attenuation structures as dolomite crystals, which occur in some Doushantuo fossils, cannot account for the structures. Dolomite crystals are uniformly grey in tomograms and completely different in appearance to the structures in question. It is also unlikely that the structures preserve a microbial infestation of the organisms. This scenario could possibly account for the structures in *Sinocyclocyclicus*, but not those in *Ramitubus*, which are organized in regular layers. Furthermore, infesting bacteria do not typically show the facetted morphology of the structures seen in the fossils, even when they form very dense biofilms that replicate subcellular anatomy (e.g. fig. 7 in [45]). The structures are therefore best interpreted as part of the anatomy of the tubular fossils. They most likely represent either cells or subcellular structures, as discussed below.

### (a) Cyanobacteria affinity

Comparisons have been drawn between the tubular taxa, especially *Sinocyclocyclicus*, and cyanobacteria [27]. Some cyanobacteria have alternating complete and incomplete cross walls resembling those seen in *Sinocyclocylicus* (e.g. *Oscillatoria*, see fig. 4 of [27]). In cyanobacteria, cross walls occur as boundaries between cells, and partial cross walls occur as cells are undergoing division. Under this interpretative model, the compartments in the fossils would be interpreted as cells. This is difficult to reconcile with the rigid and possibly even mineralized nature of the walls in *Sinocyclocyclicus* and *Quadratitubus*. In addition, it would require the taxa with incomplete cross walls to be preserved only when they are undergoing cell division. The structures within these compartments would have to be interpreted as subcellular features of cyanobacterial anatomy. Cyanobacteria have few comparable subcellular structures, however, as they lack organelles and the structures they do possess in the cytoplasm tend to be much smaller. For example, bacterial micro-compartments are usually around 100–200 nm in diameter. It is unlikely that the structures formed by degradation of cytoplasm. Cells containing degraded cytoplasm in other specimens in the deposit have a characteristic clotted fabric throughout [9], whereas the compartments in this specimen are occupied by distinct ovoid structures. Moreover, there are additional lines of evidence that cast doubt on a cyanobacterial interpretation for these fossils. The compartments have a much larger diameter—up to 270 µm in *Sinocyclocyclicus* and 625 µm in *Ramitubus* [25]—than living cyanobacteria, which rarely exceed 50 µm in diameter. They have a much smaller height to width ratio than modern cyanobacterial cells [25], though there is a general trend in extant cyanobacteria for wider cells to be thinner [46]. The cellular clusters present in *Crassitubus* and, perhaps, *Ramitubus*, represent a level of complexity not seen in bacteria; if they are a part of the organism's anatomy, they would also be incompatible with this interpretation. Finally, the cyanobacterial interpretation implies growth by intercalation, which is inconsistent with evidence for terminal growth, at least in *Ramitubus* [25].

### (b) Metazoan, eumetazoan or cnidarian affinity

A second prominent hypothesis is that the tubular taxa are stem-group eumetazoans or stem-group cnidarians. This is based largely on the comparison between the cross walls and the tabulae of the tabulate corals [12,19,25]. In addition, if the Doushantuo tubular taxa are indeed closely related to one another, then the combination of characters in the four genera—tetraradial symmetry (in *Quadratitubus*), a longitudinal ridge (in *Crassitubus*) and cross walls (in all four genera)—can be used to add some support for a cnidarian affinity [25]. Although none of these characters are unique to cnidarians, they are all found in this group and are relatively rare in other candidate groups like algae and cyanobacteria. *Ramitubus* has been considered to be the strongest candidate animal of the four genera [25] as it has regular dichotomous branching and cross walls, as well as possible nested side walls in some specimens [6]. However, the structures we have observed between the cross walls of *Sinocyclocyclicus* and *Ramitubus* are difficult to reconcile with a cnidarian body plan. The tabulae of tabulate corals, to which the cross walls have been compared, represent the successive bases of the chamber in which the polyp lived, with the polyp living above the last-formed cross wall. This model therefore predicts that the chambers will contain enough space to house the polyp and that chambers that are no longer occupied will be empty or filled with diagenetic cement, as seen in tabulate corals. For this reason, the presence of either cells or subcellular inclusions between the walls is incompatible with the coral interpretation. Alternative interpretations of the structures between the cross walls of *Sinocyclocyclicus* as degraded cytoplasm, considered unlikely for reasons outlined above, would also be incompatible with the cnidarian model for the same reason. An interpretation of these structures as infesting bacteria, again considered unlikely, would also be hard to reconcile with a cnidarian model. There would be little of nutritional value in this region, and for this reason infesting and symbiotic microorganisms are closely associated with the polyp in living cnidarians [47]. An interpretation of these structures as artefacts produced by taphonomic or diagenetic processes could be compatible with this interpretation but, as discussed above, this model cannot account for the key features of the structures.

A position elsewhere in Metazoa is unlikely because in animals with complete cross walls, the organism tends to live in the final chamber. If the spherical clusters of cells are part of the anatomy of the organism, they could potentially be interpreted as an organism that added successive chambers in a mode of growth comparable to that of tabulate corals. However, such a mode of growth is difficult to reconcile with the biological structures preserved in the spaces between cross walls. The cellular clusters in *Crassitubus* must have been in contact with several increasingly incomplete cross walls at the same time, which is also in contrast to a cnidarian growth pattern. Moreover, these clusters bear no resemblance to extant cnidarian polyps. Rejection of crown-Cnidaria characters does not allow for a stem-Cnidaria affinity as there remains no evidence for a total-group Eumetazoa or animal affinity.

### (c) Algal affinity

Another possibility is that the tubular taxa have an affinity with one of the various clades of algae. Under this model, one possible interpretation of the compartments would be as cells, and the structures within could represent preserved organelles, such as chloroplasts or lipid droplets, or other intracellular structures, such as starch grains. The pyrenoids within some chloroplasts can bear a particularly close resemblance to the fossil structures, though pyrenoids are typically arranged peripherally within cells to avoid self-shading rather than throughout the volume. It is worth noting that multiple intracellular inclusions have been observed in some embryo-like specimens from Doushantuo [9,23,43] and interpreted as lipid droplets or yolk granules. These are preserved in a low X-ray attenuation phase that is broadly similar to that of the present structures. This suggests that a subcellular interpretation is plausible, though it is difficult to distinguish between the different possible interpretations of the structures.

Alternatively, the structures themselves could be interpreted as preserved algal cells, which is consistent with their regular organization into single layers (in *Sinocyclocyclicus*) or multiple layers (in *Ramitubus*). The structures also have a mode of preservation closely comparable to undisputed cells in the same biota. Cells in some Doushantuo algal specimens are preserved in a similar style, with a low-attenuation phase in the cell interior, surrounded by a high-attenuation phase that fills the spaces between the cells (e.g. fig. 2*a* of [23]). This high-attenuation phase between the structures has the characteristics of late stage mineralization and most likely represents a secondary diagenetic mineralization, rather than mineralization that replaces an original biological structure such as a cell wall. The fossilized ‘wall’ is not of constant thickness, but rather fills spaces between the structures.

Although we consider that the structures are best interpreted as part of the anatomy of the tubular organisms, we cannot currently distinguish between a cellular interpretation and various subcellular interpretations, not least because there are apparent difficulties with each interpretation. The problems with an interpretation of the compartments as cells are discussed above in connection with a possible cyanobacteria interpretation. In particular, there are difficulties understanding the rigid nature of walls in cells undergoing division and why cells would only be preserved when partway through cell division in some taxa. On the other hand, there are also some difficulties with an interpretation of the structures within the compartments as cells. We know of no extant taxa in which undifferentiated cells are enclosed within compartments, which they presumably created.

Nonetheless, there are broadly analogous algal taxa regardless of how the structures between the cross walls are interpreted, although it is difficult to place the fossils in any particular group with confidence. Under an interpretation of these structures as cells, some red algae, such as the coralline alga *Amphiroa* [48], provide a possible comparison as they produce cross walls between rows of cells. However, they differ from *Sinocyclocyclicus* in that their cell walls are mineralized in their entirety. As the structures between the cross walls of *Sinocyclocyclicus* are unlikely to have been mineralized, it seems more likely that the walls were produced by the organism. If, on the other hand, the compartments are interpreted as individual cells, then green algae such as *Spirogyra* provide an analogue, as the pyrenoids in their chloroplasts can form closely packed structures that resemble those of the fossils.

## 6. Conclusion

We find that the patterns of deformation observed in the Doushantuo tubular fossils suggest that the cross walls of *Sinocyclocyclicus* and *Quadratitubus* were more rigid than those of *Ramitubus* and *Crassitubus*. *Ramitubus* and *Crassitubus* specimens preserve enigmatic cellular clusters at terminal positions in the tubes. Specimens of *Sinocyclocyclicus* and *Ramitubus* have biological features that might be cellular tissue or subcellular structures filling the spaces between the cross walls. Either interpretation of these features is incompatible with an interpretation as a cnidarian-like animal.

The Doushantuo tubular fossils are rare examples of taxa that have been widely accepted as Neoproterozoic eumetazoans. However, the new data presented here do not support this affinity. As a result, these fossils do not reduce the gap between molecular clock predictions for the divergence of animals and the fossil evidence. Resolving this issue will require improvements in prediction of the nature of early animals based in comparative anatomy and comparative developmental biology, and in resolving phylogenetic conundrums such as monophyly versus paraphyly of sponges, and the phylogenetic position of ctenophores. However, devising improved tests of the affinities of fossils that remain in contention as the earliest animals will also be vital.

## Supplementary Material

Supplementary figures 1-3

## Data accessibility

The datasets supporting this article are available at http://dx.doi.org/10.5523/bris.13g2svxco0zml1lmzde60hfqlf.
