# Supplementary material for: Critical appraisal of tubular putative eumetazoans from the Ediacaran Weng'an Doushantuo biota
Source: Proc Biol Sci. 2015 Aug 7;282(1812):20151169. doi: 10.1098/rspb.2015.1169 (PMC4528530; doi:10.1098/rspb.2015.1169)
Supplement: Supplementary figures 1-3 [file rspb20151169supp1.pdf]

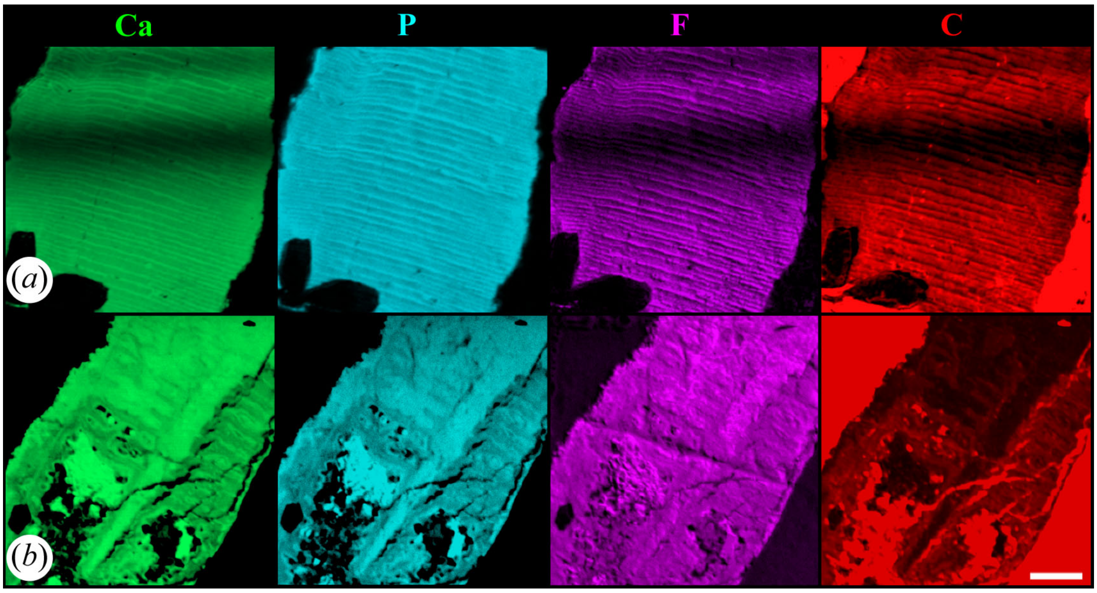

Supplementary figure 1. Electron probe microanalysis (EPMA) maps of tubular fossils. Each map shows the relative abundance of the element, with brighter tones representing greater abundance. Regions of high and low X-ray attenuation have similar chemical signals to those observed in other Weng'an fossils by [23]. (*a*) *Sinocyclocyclicus* specimen X 5330 (note that the dark horizontal bands in the Ca, F and C maps are artefacts); (*b*) *Ramitubus* specimen X 5326. Scale bar: 50 μm.

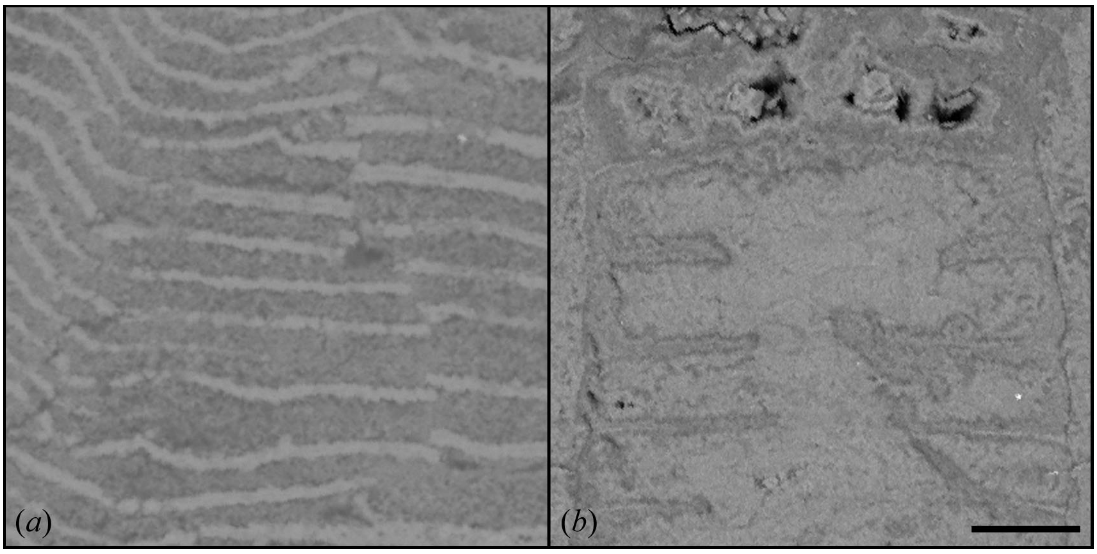

Supplementary figure 2. Backscattered electron (BSE) images of the cross walls of Doushantuo tubular fossils. (*a*) *Sinocyclocyclicus* specimen X 5330 showing walls that are preserved in a high atomic number phase and are broken and displaced providing evidence of brittle fracture; (*b*) *Ramitubus* specimen X 5326 showing irregular regions that are surrounded by incomplete cross walls and filled by void-filling cement providing evidence of organic degradation of the cross walls. Scale bar: (*a*) 10 µm; (*b*) 20 µm.

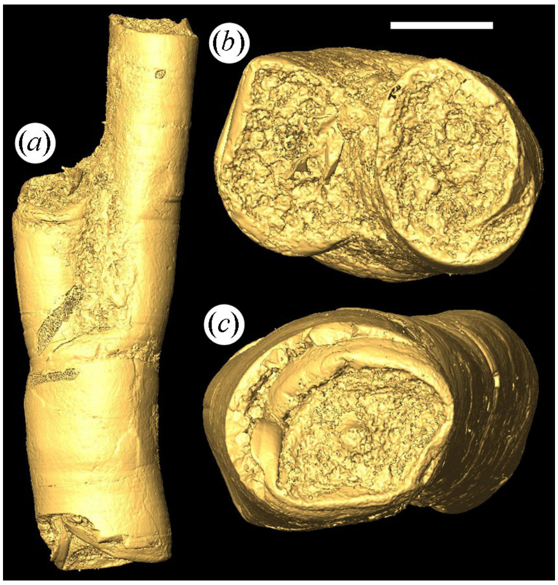

Supplementary figure 3. Surface models of *Ramitubus* specimen X 5325 based on SRXTM data showing tearing of the specimen. (*a*) lateral view; (b) ventral view; (c) dorsal view. Scale bar: (*a*) 227 µm; (*b*-*c*) 135 µm.
